# Pulmonary Mucormycosis in a Poorly Controlled Diabetic Patient

**DOI:** 10.7759/cureus.24932

**Published:** 2022-05-12

**Authors:** Mafalda Duarte, Vasco Tiago, Abelcineyd Camble, Raquel Sousa, Fernando Aldomiro

**Affiliations:** 1 Internal Medicine, Hospital Professor Doutor Fernando Fonseca, Amadora, PRT

**Keywords:** pulmonary mucormycosis, severe pneumonia, influenza b, diabetic ketoacidosis (dka), diabetes mellitus(dm)

## Abstract

Mucormycosis is a severe fungal infection that occurs mainly in immunocompromised patients. It is characterized by several syndromes with rhino-orbito-cerebral and pulmonary involvement.

We report the case of a 49-year-old patient admitted for diabetic ketoacidosis and Influenza B pneumonia nonresponsive to treatment, which was later diagnosed with pulmonary mucormycosis. After correct diagnosis and appropriate treatment with isavuconazole, the patient had a favorable evolution, which reinforces the importance of an accurate diagnosis.

## Introduction

Human mucormycosis, previously known as zygomycosis, is a rare fungal infection that affects particularly immunocompromised patients. The most commonly found genera in human infections are *Rhizopus*, *Mucor*, and *Rhizomucor*. Their broad and irregular hyphae infect humans by inhalation of spores [1].

About 40% of cases have a rhino-orbital or cerebral presentation, especially in diabetic patients [1]. Other forms of presentation such as pulmonary, primary cutaneous (mostly in premature infants), gastrointestinal and disseminated can also occur, especially in severely immunocompromised patients [2].

Several studies reported that the types of immunosuppression more commonly associated with this infection are: diabetes mellitus (particularly with ketoacidosis), treatment with glucocorticoids, hematopoietic cell and solid organ malignancies, iron overload, Acquired Immunodeficiency Syndrome (AIDS), intravenous drug use, trauma/burns and extreme malnutrition [3-6].

The literature shows that the association of mucormycosis with hyperglycemia seems to be related to the impaired ability of neutrophils to destroy the *Mucorales *hyphae in this specific situation. It is also known that the *Mucorales *use iron as a growth factor and that in hyperglicemic patients the availability of free iron is increased [1, 4]. 

The differential diagnosis with aspergillosis is challenging but they are two different infectious entities that do not respond to the same treatment: mucormycosis has a natural resistance to voriconazole, which is the first-line treatment for aspergillosis [1].

## Case presentation

A 49-year-old Black man was found unconscious and brought to the Emergency Department. He had a known medical history of type 2 diabetes mellitus with several macro- and microvascular complications. The patient had abandoned all his medical follow-up five years earlier and was not taking any medication. His last glycated hemoglobin value, five years before the admission, was 10.5%.

Upon hospital admission, he was stuporous (Glasgow Coma Score [GCS] of 11), with signs of peripheral hypoperfusion, and he was polypneic under 10 litres/minute of supplemental oxygen. The detailed laboratory results are presented in Table 1 and show metabolic acidosis, hyperglycemia with hyperketonemia, and severely increased inflammatory biomarkers. Also noteworthy is the high glycated hemoglobin value of 21.7%.

**Table 1:** Laboratory results pCO2: partial pressure of carbon dioxide, pO2: partial pressure of oxygen, HCO3: bicarbonate, mmol/L: millimoles per litre, mEq/L: milliequivalents per litre, WBC: white blood cell, μl/L: microliter per litre, mg/dL: milligrams per decilitre, RCP: reactive chain protein, Cr: creatinine, HbA1C: glycated hemoglobin, HIV: human immunodeficiency virus.

Laboratory studies	Results	Reference values
pH	6.9	7.35-7.45
pCO2 (mmol/L)	17	35-45
pO2 (mmol/L)	200	80-100
HCO3 (mmol/L)	3.1	22-26
Anion Gap (mEq/L)	37	8-12
Glycemia (mg/dL)	644	<120
Ketonemia (mmol/L)	4.2	<0.6
WBC count (μl/L)	27.1 x 10^9^	4.5-10
Neutrophils (%)	92	50-70
RCP (mg/dL)	20	<0.5
Cr (mg/dL)	1.44	0.7-1.2
HbA1C (%)	21.7	<6.5%
HIV serology	negative	-
Blood cultures	negative	-

The chest X-ray revealed extensive multilobar infiltrates, suggestive of interstitial pneumonia. He also tested positive for the Influenza B virus (diagnosis by polymerase chain reaction). The diagnosis of Influenza B pneumonia with bacterial superinfection and diabetic ketoacidosis was assumed, and guided pharmacological therapy was started. The ketoacidosis was corrected with insulin and intravenous fluids with progressive clinical and analytical improvement. Antiviral treatment with oseltamivir and empirical antibiotic treatment with ceftriaxone 2 grams and clarithromycin 500 mg (milligrams) two times per day, were also initiated.

**Figure 1:**
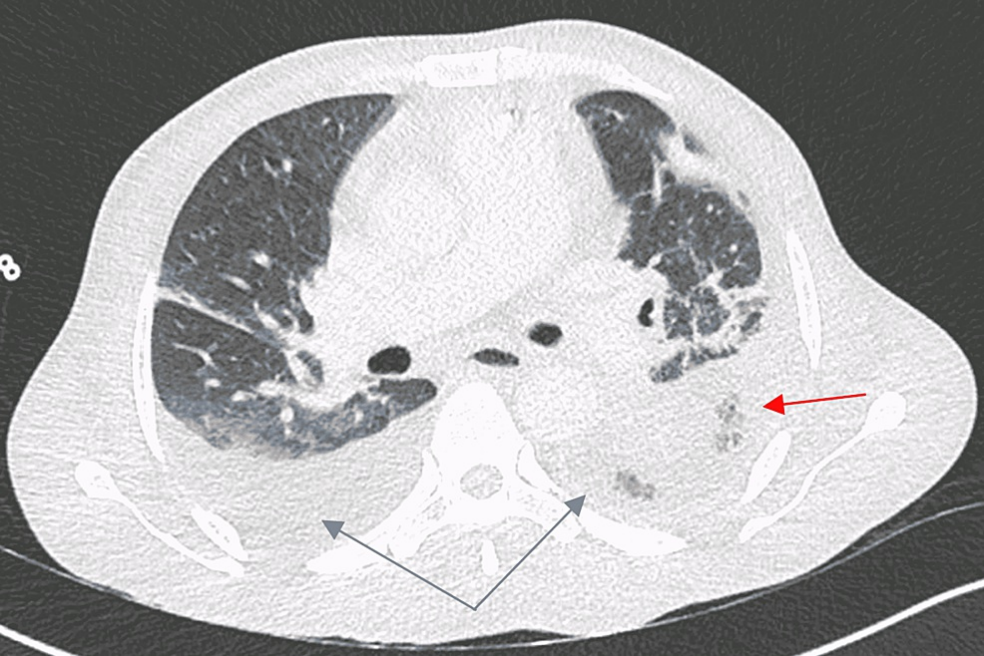
Thorax CT scan showing bilateral pleural effusion and parenchymal densification of the left lung Gray arrows: bilateral pleural effusion; red arrow: parenchymal densification of the left lung.

After a few days, he evolved poorly with higher need for supplemental oxygen and no clinical response to the antiviral treatment and to the antibiotics. A chest computerized tomography (CT) scan was performed which revealed bilateral pleural effusion, extensive parenchymal densification, and adjacent collapse of the inferior left lung lobe (Figure 1).

**Figure 2:**
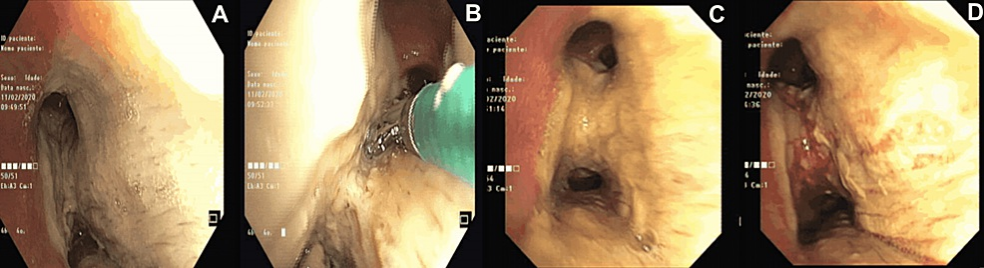
Bronchoscopy images of white adherent plaques suggestive of fungal infection

Considering all the CT scan findings and the assumption of a refractory pneumonia in an immunosuppressed host, a bronchoscopy was performed revealing several white adherent plaques impossible to remove (Figure 2).

The plaques were highly suggestive of fungal infection and a brochoalveolar lavage was performed followed by empirical treatment with fluconazole. However, while on empirical therapy, the patient's condition continued to deteriorate, with worsening respiratory failure and large volume parapneumonic empyema, requiring an intensive care unit stay for three days. At the intensive care unit, he was stabilized and a thoracentesis was performed. He improved slightly and was again transferred to the internal medicine department.

The bronchoalveolar lavage previously performed revealed evidence of yeasts and hyphae suggestive of mucormycosis. After consultation with the Infectiology Department, isavuconazole therapy was started: an initial dose of 200 mg three times per day, for 48 hours, followed by a maintenance dose of 200 mg per day.

The patient had a favorable clinical course with progressive laboratorial and radiological improvement, and respiratory failure resolution. He was discharged to a rehabilitation institution at the 42nd day of his hospital stay, with no need of supplemental oxygen, complying with the six-week course of isavuconazole therapy.

**Figure 3:**
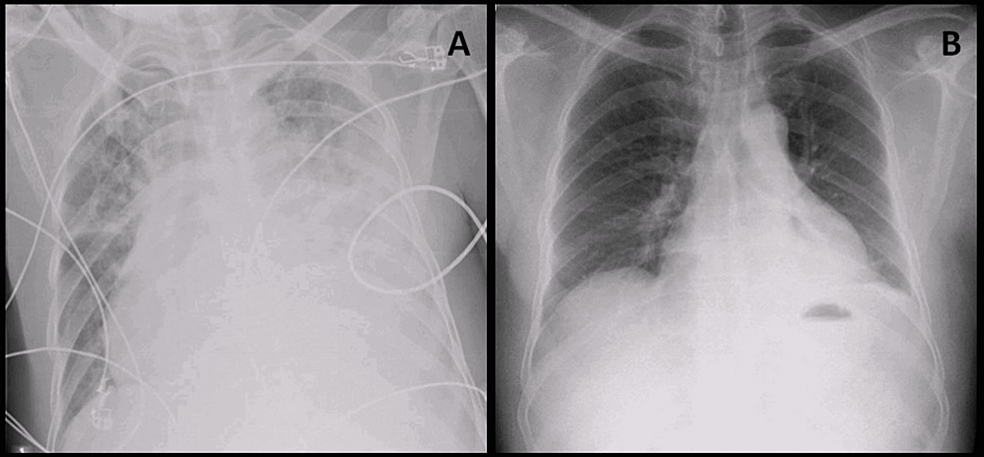
Imagiological evolution A: Admission chest X-ray; B: chest X-ray 2 months after finishing treatment.

Two months after hospital discharge, the patient was evaluated and did not have respiratory symptoms and imagiological resolution (Figure 3). After this appointment, he was lost to follow up.

## Discussion

Mucormycosis is a challenging disease with mortality rates as high as 87 percent in patients with pulmonary mucormycosis. Currently, we know that early diagnosis and initiation of appropriate treatment increase survival from approximately 40% to 80% [5].

Concerning the diagnostic approach, we are aware that biopsy cultures are positive in only 50% of cases whereas beta-D-glucan and galactomannan antigenemia are frequently negative. Several studies showed that the internal transcribed spacer (ITS) region is the most widely sequenced DNA region for fungi, and it is recommended as a first-line method for species identification of *Mucorales* [5,6]. Unfortunately, this specific diagnostic test is not widely available. Because there are no specific diagnostic criteria or a well-known typical presentation, its diagnosis requires a high level of clinical suspicion.

With respect to the treatment, the first line of treatment is liposomal amphotericin B, despite its adverse effects such as nephrotoxicity [1,7]. Recent studies showed that isavuconazole has a similar efficacy and a better safety profile [7], the reason why it was used in this case, after discussion with the Infectiology Department.

## Conclusions

Mucormycosis is a life-threatening infection that affects mostly immunocompromised hosts. Its early diagnosis requires a high level of clinical suspicion and great awareness of mucormycosis' associated risk factors, such as diabetes mellitus. A multidisciplinary collaboration between pathologists, mycologists, and the other attending physicians is also indispensable to prompt management of the disease. Early diagnosis allows proper treatment and improves survival rates.

## References

[1] Point S, Gabriel F, Bégueret H (2018). Tumor shape pulmonary mucormycosis associated with sinonasal aspergillosis in a diabetic patient. Med Mycol Case Rep.

[2] Roden MM, Zaoutis TE, Buchanan WL (2005). Epidemiology and outcome of zygomycosis: a review of 929 reported cases. Clin Infect Dis.

[3] Petrikkos G, Skiada A, Lortholary O, Roilides E, Walsh TJ, Kontoyiannis DP (2012). Epidemiology and clinical manifestations of mucormycosis. Clin Infect Dis.

[4] Lee FY, Mossad SB, Adal KA (1999). Pulmonary mucormycosis: the last 30 years. Arch Intern Med.

[5] Walsh TJ, Skiada A, Cornely OA (2014). Development of new strategies for early diagnosis of mucormycosis from bench to bedside. Mycoses.

[6] Feng J, Sun X (2018). Characteristics of pulmonary mucormycosis and predictive risk factors for the outcome. Infection.

[7] Jenks JD, Salzer HJ, Prattes J, Krause R, Buchheidt D, Hoenigl M (2018). Spotlight on isavuconazole in the treatment of invasive aspergillosis and mucormycosis: design, development, and place in therapy. Drug Des Devel Ther.

